# Comparison of Cinematic Rendering and Computed Tomography for Speed and Comprehension of Surgical Anatomy

**DOI:** 10.1001/jamasurg.2019.1168

**Published:** 2019-05-29

**Authors:** Moustafa Elshafei, Johannes Binder, Justus Baecker, Maximilian Brunner, Michael Uder, Georg F. Weber, Robert Grützmann, Christian Krautz

**Affiliations:** 1 Department of Surgery, Universitätsklinikum Erlangen, Friedrich-Alexander-Universität Erlangen Nürnberg, Erlangen, Germany; 2 Institute of Radiology, Universitätsklinikum Erlangen, Friedrich-Alexander-Universität Erlangen Nürnberg, Erlangen, Germany

## Abstract

**Importance:**

Three-dimensional (3-D) volume rendering has been shown to improve visualization in general surgery. Cinematic rendering (CR), a novel 3-D visualization technology for postprocessing of computed tomographaphy (CT) images, provides photorealistic images with the potential to improve visualization of anatomic details.

**Objective:**

To determine the value of CR for the comprehension of the surgical anatomy.

**Design, Setting, and Participants:**

This preclinical, randomized, 2-sequence crossover study was conducted from February to November 1, 2018, at University Hospital of Erlangen, Germany. The 40 patient cases were evaluated by 18 resident and attending surgeons using a prepared set of CT and CR images. The patient cases were randomized to 2 assessment sequences (CR-CT and CT-CR). During each assessment period, participants answered 1 question per case that addressed crucial issues of anatomic understanding, preoperative planning, and intraoperative strategies. After a washout period of 2 weeks, case evaluations were crossed over to the respective second image modality.

**Main Outcomes and Measures:**

The primary outcome measure was the correctness of answers. Secondary outcome was the time needed to answer.

**Results:**

The mean (SD) interperiod differences for the percentage of correct answers in the CR-CT sequence (8.5% [7.0%]) differed significantly from those in the CT-CR sequence (−13.1% [6.3%]) (*P* < .001). The mean (SD) interperiod differences for the time spent to answer the questions in the CR-CT sequence (−18.3 [76.9] seconds) also differed significantly from those in the CT-CR sequence (52.4 [88.5] seconds) (*P* < .001). Subgroup analysis revealed that residents as well as attending physicians benefitted from CR visualization. Analysis of the case assessment questionnaire showed that CR added significant value to the comprehension of the surgical anatomy (overall mean [SD] score, 4.53 [0.75]). No carryover or period effects were observed.

**Conclusions and Relevance:**

The visualization with CR allowed a more correct and faster comprehension of the surgical anatomy compared with conventional CT imaging, independent of level of surgeon experience. Therefore, CR may assist general surgeons with preoperative preparation and intraoperative guidance.

## Key Points

**Question:**

Does the use of cinematic rendering improve the comprehension of the surgical anatomy?

**Findings:**

In this German preclinical randomized crossover study, visualization with cinematic rendering allowed a more correct and faster comprehension of the surgical anatomy compared with conventional computed tomography independent of the level of surgical experience.

**Meaning:**

Cinematic rendering is a tool that may assist general surgeons with preoperative preparation and intraoperative guidance through an improved interpretation of computed tomography imaging data.

## Teaser

This German preclinical randomized crossover study assesses the anatomical understanding of resident and attending surgeons after reviewing patient cases by cinematic rendering and by computed tomography.

## Introduction

In many fields of general surgery (eg, hepatopancreatobiliary surgery), accurate knowledge and understanding of the patient’s anatomy with all details (eg, vasculature) is the key requirement in surgical decision making. The interpretation of complex anatomy based on conventional cross-sectional imaging is difficult and susceptible to errors because it requires advanced spatial reasoning abilities. Misinterpretation of imaging data corrupts preoperative decision making and the designated surgical approach, which, in turn, may jeopardize patient outcomes. Virtual 3-dimensional (3-D) reconstruction techniques have been developed to overcome this subjective limitation, ultimately facilitating comprehension of patient anatomy.^1,2,3^ However, conventional volume-rendering techniques provide images of limited quality that do not allow an adequate evaluation of complex intra-abdominal structures, such as the liver. In this regard, a more photorealistic visualization may facilitate image interpretation and improve the comprehension of the surgical anatomy.^4,5^

In 2016, a new technique for 3-D visualization of cross-sectional image data called cinematic rendering (CR) was introduced.^6,7,8^ Cinematic rendering is a physically based volume-rendering method that works with random sampling computational algorithms. Different light maps and transfer functions are used to generate a realistic depiction of medical imaging data.^8^ This enhanced simulation of the paths of light rays enables CR to provide images with higher quality compared with conventional volume-rendering techniques.^4,5,7,8,9,10,11,12,13^

In our study, we assessed the value of CR in the comprehension of surgical anatomy. Using a customized workstation for postprocessing and real-time acquisition of CR images, we compared computed tomography (CT) with CR imaging using objective and subjective assessment questionnaires.

## Methods

### Cinematic Rendering: Technical Background

Cinematic rendering is a physically based volume-rendering technique. This crossover study used a Monte Carlo path-tracing method to compute the interaction of photons with the scanned patient data.^6^ This path-tracing method was first deployed in computer animation programs by the entertainment industry.^7^ This rendering method works with data retrieved from conventional CT or magnetic resonance scans. Hence, the image quality is determined by the original resolution and increases with the number of light paths that are traced. The use of high-dynamic-range–rendering light maps for illumination and the real-time computation of complex lighting effects produce a photorealistic depiction of the image data (Video 1).

**Video 1.** Cinematic Rendering Animation of Patient Case 14.

The Cinematic Rendering for Surgery application used in this study was developed in a multistep innovation process in a collaboration between Siemens Healthineers and the Department of Surgery of the University Hospital of Erlangen. This prototype is still used only for research purposes and is not yet approved for clinical use. Digital Imaging and Communications in Medicine (DICOM) data can be uploaded directly into the application without conversion. The interface displays the patient’s name and sex, imaging modality, type of radiologic protocol (eg, CT for pancreatic imaging), slice thickness, and number of images with a small, multiplanar reformat preview in the axial plane on the side. The prototype version has multiple options to manipulate the image, and 2 different display presets can be chosen. The image can be sliced with 3 clipping planes. Because of the importance of vasculature in surgery, there is an option to virtually show the vessels in front of the sectional plane, and multiplanar reformat images can be overlaid. Lighting (Brightness), window-levelling (Range), and window center (Anatomy) can be selected. The images are rendered in real time, and the quality depends on the resolution of the original image.

### Participants and Patient Cases

This study followed the Consolidated Standards of Reporting Trials (CONSORT) reporting guideline. The medical ethics review committee of the University Hospital of Erlangen (University of Erlangen-Nürnberg, Germany) approved the study protocol. Informed consent was deemed unnecessary because we used completely anonymized imaging data. We randomly asked general surgery residents and attending surgeons from our department, the Department of Surgery of the University Hospital of Erlangen, to participate in this study. Nine resident surgeons and 9 attending surgeons agreed to participate in this study. Resident surgeons who participated were in postgraduate year 2 to 6. None of the participants had previous experience with volume-rendering techniques.

Using an electronic database, we identified patient cases who were treated or followed up for hepatopancreatobiliary tumors at the Department of Surgery in the University Hospital of Erlangen, Germany, from January 1, 2015, through January 1, 2017. Subsequently, we selected 40 patients who had undergone a high-resolution CT of the abdomen at any time during their course of treatment. Only anonymized DICOM data from CT images with a maximum slice thickness of 1 mm were used.

**Figure 1.**
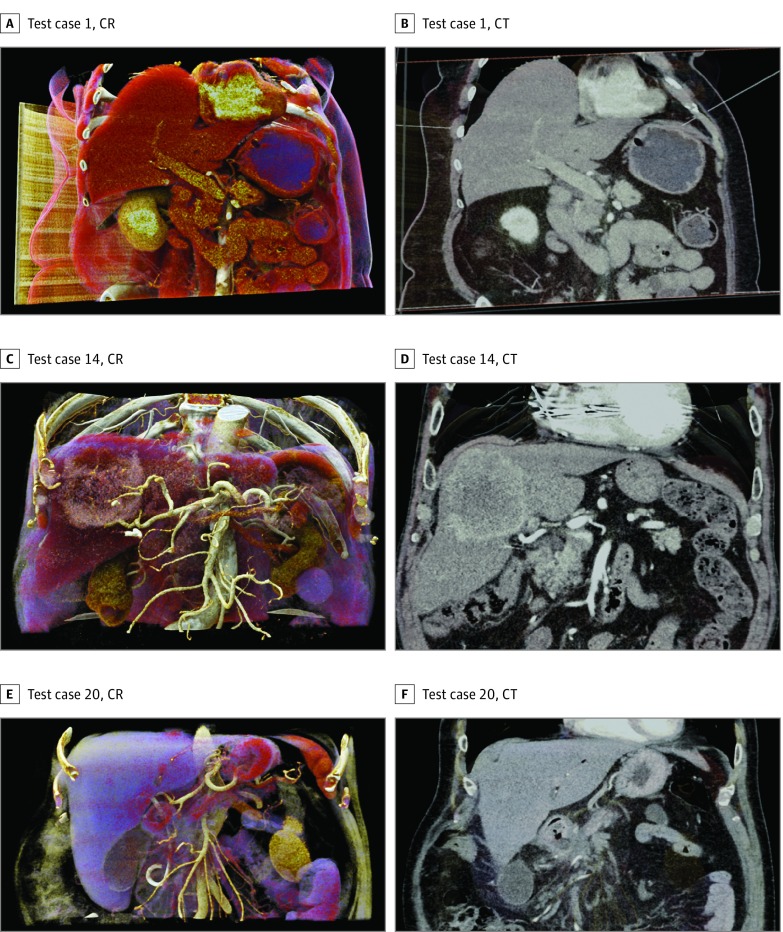
Example Starting Images and Questions From Patient Cases Cinematic rendering (CR) and computed tomography (CT) images. A and B, test case 1 (Does the pancreatic head tumor have contact to the superior mesenteric artery?); C and D, test case 14 (Is the tumor supplied by vessels from the left hepatic artery?); and E and F, test case 20 (Show and name the arterial supply of the left liver lobe).

For each patient case, 1 question addressing crucial issues of anatomic understanding, preoperative planning, and intraoperative strategies was formulated (eTable 1 in the Supplement). These questions had to be answered by using either CR visualization or conventional CT imaging. The correct answers were predefined in the study protocol. Using the preset function of the application, CR and CT imaging packages were automatically generated in a standardized fashion for each patient case. We then set up starting images depicting the area of interest in 2 different planes for each imaging modality to speed up the evaluation process (Figure 1). The prepared imaging packages were used by all participants.

### Study Design and Assessment

**Figure 2.**
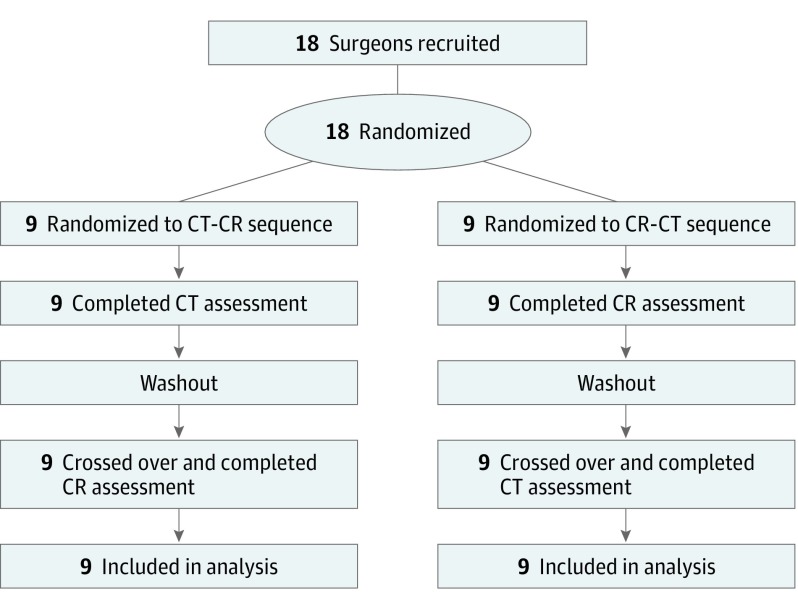
Study Design CR indicates cinematic rendering; CT, computed tomography.

This preclinical study with a randomized 2-sequence crossover design was conducted from February to November 1, 2018, at the Department of Surgery in the University Hospital of Erlangen, Germany (Figure 2). For each participant, the selected patient cases were randomized either to a CR-CT sequence or a CT-CR sequence. The number of cases within the 2 sequence groups were balanced in regard to the experience of the surgeon (resident vs attending). The participants conducted their evaluations at separate time points in the presence of 1 interviewer (C.K.).

After an introduction to the CR for surgery application, all participants were allowed to acclimate themselves to handling the hardware and software components. In addition, 2 separate patient cases were used to simulate the flow of the study, which enabled each participant to familiarize himself or herself with the objective assessments and self-assessment questionnaires.

After this orientation, all participants completed the first assessment of 40 prepared patient cases according to the assigned image modality. After a washout period of at least 2 weeks, all cases were crossed over to the alternate imaging modality for a second assessment.

For an objective assessment, all surgeons had to answer the predefined questions (1 per patient case). The interviewer tracked and documented the outcome measures. There was no time restriction. The primary outcome was the correctness of the answers. The secondary outcome was the time needed to give the answer.

We applied a case assessment questionnaire to rate participants’ perception of the advantage of using CR compared with conventional CT images in each CR assessment. This questionnaire consisted of 4 categories: comprehension of general anatomy, comprehension of vascular anatomy, comprehension of parenchymal anatomy, and comprehension of spatial relationships (eTable 2 in the Supplement). Moreover, a general assessment questionnaire we designed was applied at the beginning of the first assessment period and at the end of the second assessment period. This 9-item questionnaire was set up to explore a possible benefit of CR in 4 categories: general decision making, interdisciplinary decision making, intraoperative guidance, and informed consent discussions (eTable 3 in the Supplement). Both questionnaires were based on a typical 5-point Likert scale ranging from 1 for strongly disagree to 5 for strongly agree.

### Statistical Analysis

Results are presented as mean (SD) or as frequency data. Time to answer and the percentage correct were computed as treatment effects, period effects, and carryover effects by the method reported by Hills and Armitage^14^ for 2-period crossover clinical trials. These data were analyzed using the independent *t* test to evaluate between-group differences of the 2 sequence groups. For the interperiod difference, the difference of assessment period 1 and assessment period 2 was computed. Differences between resident and attending physicians were analyzed with the Fisher exact test (questionnaire scores) or with the independent *t* test (time to answer and percentage of correctness).

 Statistical significance was determined as 2-sided *P* < .05. The analysis was conducted using SPSS, version 20 (SPSS Inc).

## Results

### Objective Assessment

**Figure 3.**
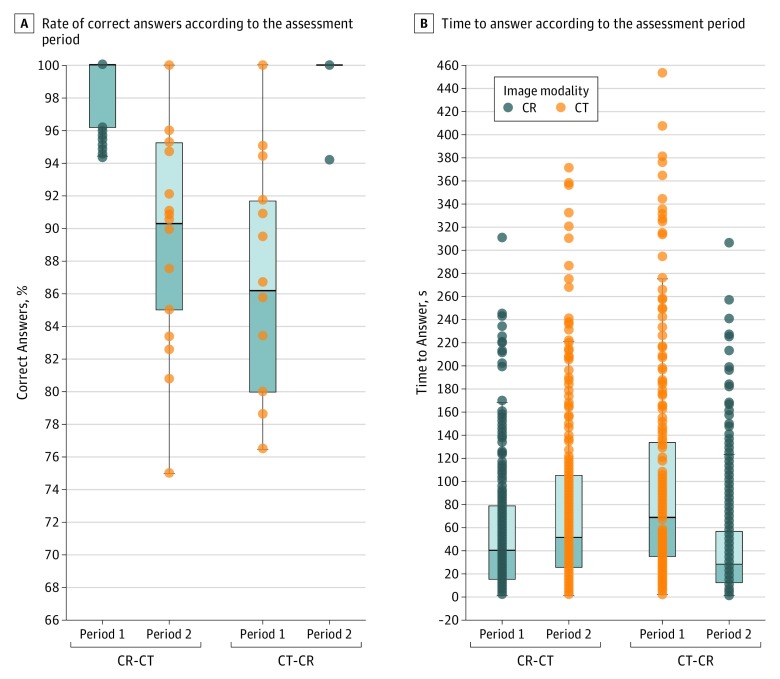
Association of Cinematic Rendering (CR) With Correctness of Answers and Time to Answer A, Rate of correct answers. B, Time needed to answer according to the sequence. CT indicates computed tomography.

Eighteen surgeons completed a total of 720 case evaluations. Cinematic rendering visualization was associated with a better anatomic understanding compared with conventional CT imaging. For the CR-CT sequence, the overall mean (SD) percentage of correct answers for CR assessment was 98.7% (2.2%); for CT assessment, 90.2% (7.0%); and for mean (SD) difference over time, 8.5% (7.0%). For the CT-CR sequence, the overall percentage of correct answers for CT assessment was 86.6% (6.6%); for CR assessment, 99.7% (1.4%); and for mean difference over time, –13.1% (6.3%); *P* < .001 by independent *t* test (Figure 3B). This association was also detectable in the subgroups of resident and attending surgeons (eTable 4 in the Supplement). The percentage of correct answers did not differ significantly between resident and attending surgeons for the CT assessment (86.9% [6.6%] vs 89.9% [7.2%]; *P* = .21) and for the CR assessment (99.2% [1.8%] vs 99.1% [2.0%]; *P* = .90).

Mean time spent by all participants with the CR assessment was significantly shorter than with the CT assessment. For the CR-CT sequence, the mean (SD) time for the CR assessment was 56.6 (54.6) seconds; for CT assessment, 75.0 (69.1) seconds; and for mean (SD) difference over time, –18.3 (76.9) seconds. For the CT-CR sequence, the mean (SD) time for the CT assessment was 95.1 (83.7) seconds; for CR assessment, 42.7 (48.9) seconds; and for mean (SD) difference over time, 52.4 (88.5) seconds; *P* < .001 by independent *t* test (Figure 3A). This association was still evident after stratification according to surgeon experience (resident vs attending) (eTable 4 in the Supplement). Moreover, resident surgeons needed significantly more time for their answers compared with attending surgeons, no matter which visualization modality they used (CT: residents, 103.3 [87.2] seconds vs attending surgeons, 66.8 [60.8] seconds; *P* < .001 and CR: residents, 55.7 [54.3] seconds vs attending surgeons, 43.6 [49.6] seconds; *P* = .002). However, time reduction by CR was 48% for resident surgeons and 32.5% for attending surgeons (*P* = .01). No carryover or period effects were observed.

### Case Assessment Questionnaire

**Figure 4.**
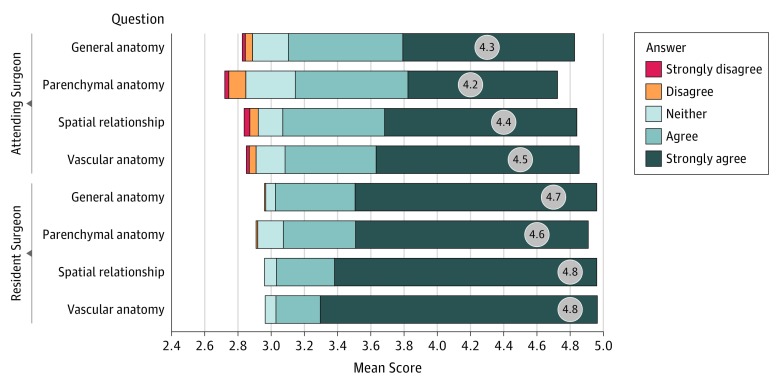
Responses to the Case Assessment Questionnaire Mean scores are depicted within the gray circles.

The ratings of the case assessment according to the different categories and surgeon experience are summarized as a Likert plot in Figure 4. In total, the self-assessment questionnaire revealed that, in most cases, participants agreed that CR is beneficial for the comprehension of the surgical anatomy (overall mean (SD) score, 4.53 [0.75]). Independent of surgeon experience, the question categories of vascular anatomy and spatial relationship received the highest scores (vascular anatomy, 4.63 [0.68]; spatial relationship, 4.58 [0.73]). The mean (SD) score for general anatomy was 4.52 (0.72) and for parenchymal anatomy, 4.39 (0.84). In all categories, the ratings of resident surgeons were significantly higher than those of the attending surgeons (eTable 5 in the Supplement).

### General Assessment Questionnaire

The responses to the general assessment questionnaire according to different categories and surgeon experience are summarized as a Likert plot in the eFigure in the Supplement. The highest scores were given for the statement “CR may help with explanations during informed consent discussions.” As in the case assessment questionnaire, ratings of resident surgeons tended to be higher than those of the attending surgeons. Only the scores for question 9 (“CR can help with explanations during informed consent discussions”) differed significantly between resident and attending surgeons (mean [SD] score; resident surgeons, 5.0 [0]; attending surgeons 4.3 (0.9); *P* = .04).

Resident surgeons rated CR as beneficial in relation to all 9 questions (mean [SD] score for each question, >3). Attending surgeons were undetermined toward a beneficial influence of CR on interdisciplinary decision making for multimodal therapy concepts (mean [SD] score for question 8: 2.9 [1.6]) and in regard to a reduction of time needed for therapeutic decisions (question 4: 3.0 [1.5]). The responses to the general assessment questionnaire did not differ significantly over time (eg, mean [SD] score for question 1: first assessment, 3.3 [1.3]; second assessment, 3.5 [1.3]; *P* = .30).

## Discussion

Our investigation showed that CR imaging reduces the time needed to answer anatomy-related questions. Most of all, the correctness of answers increased when CR was used. In regard to the complexity of our questions, these results demonstrate that CR helps to transfer complex anatomical information to clinicians. Recently, Marconi et al^15^ reported that 3-D–printed models allowed the best anatomical understanding, with faster and clearer comprehension of the surgical anatomy. This study was set up to validate the preoperative use of 3-D–printed anatomical models in comparison with conventional CT imaging and volume-rendering visualizations. Both 3-D–printed anatomical models and conventional volume-rendering visualizations significantly increased the correctness of answers and the time spent for evaluations compared with conventional CT imaging. However, there were no significant differences between conventional volume-rendering visualizations and 3-D–printed models. The authors stated that the possibility of grasping a physical object is the most evident advantage of 3-D–printed models, allowing a mental reconstruction and memorization of the anatomy. Because there is a substantial improvement of depth perception in CR reconstructions, we think that CR, in contrast to conventional volume rendering, offers similar advantages. At the same time, CR visualization can be easily and quickly provided via the Cinematic Rendering for Surgery application through real-time postprocessing using a computer or tablet. In addition, there is no need for a 3-D printer or radiologic validation of the virtual model that is retrieved from a series of medical images after image segmentation.

That CR visualization improves the correctness of image interpretation is an obviously important finding of this study. However, from our point of view, the time savings conferred by the use of CR are equally important, because time is a rare commodity in the daily routine of surgeons. Tools that speed up image interpretation will help to save time in a variety of situations (eg, preoperative planning, intraoperative decision making). In addition, multiparty decision making may benefit, if all participants have access to a faster assessment of volume imaging.

In the present study, we also aimed to evaluate the benefits of CR imaging in regard to the level of expertise. As expected, the time to answer was significantly greater in the resident surgeon group than in the attending surgeon group, regardless of the imaging technique. Of note, the time reduction through CR imaging was significantly greater for resident (48%) than attending surgeons (32.5%). These results show that resident surgeons benefit more than attending surgeons when using CR imaging, although the increase of correct answers did not significantly differ between resident and attending surgeons.

We applied a case assessment questionnaire to rate the participants’ perception of the advantage of using CR in regard to the understanding of the patient anatomy. Independent of their level of experience, surgeons perceived CR as beneficial for the comprehension of vascular anatomy and spatial relationship (highest mean scores). This finding is in line with previous results reporting particularly impressive visualizations of high-density and high-contrast structures such as bones or contrast-enhanced vessels by CR.^2^ In addition, these results reflect the substantial contribution of CR visualization to depth perception by complex lighting effects.

Cinematic rendering is a technique for 3-D visualization that confirms decisions through real-time acquisition of photorealistic and lifelike images from medical data. These features may allow a deployment of 3-D visualizations in new fields of application. To explore the participants’ opinions on potential future applications of CR, we included a general assessment questionnaire in our study. There was an overall agreement that CR may be helpful in regard to general and interdisciplinary decision making, intraoperative guidance, and informed consent discussions. Resident surgeons tended to have a more positive outlook on future applications of CR. In this context, we think that CR is a perfect tool to enhance anatomy education of surgical residents and medical students.^10,16^ Photorealistic images are suitable to recall and deepen knowledge of anatomy. Cinematic rendering also provides the possibility of virtual immersion in patient cases involving different variants of anatomical structures or manifestations of diseases.

### Limitations

This study has several limitations worthy of consideration. First, adaptation to the CR software may have added to response time, because CR was unfamiliar and possibly appeared more complex than conventional imaging in the first place. Although all surgeons were obliged to test the CR software with 2 additional cases before being admitted to this study, we cannot exclude the possibility that there has been a learning curve in handling the CR software during the first CR cases of this study. Second, the emphasis of this study was a general evaluation of CR imaging and not to examine differences in visualization among different tissue types. Although we conducted a subjective case assessment, we cannot draw conclusions on objective benefits of CR in regard to certain types of tissues. Third, because this is a nonclinical study, we can only speculate about the potential influence that CR imaging may have on surgeons owing to an improved comprehension of the patient anatomy. Thus, it remains to be determined whether the routine clinical use of CR may improve surgical decision making, ultimately leading to a reduction of intraoperative mistakes and an improvement in patient outcomes.^1^ In this regard, further research should focus on intraoperative guidance, including an integration of CR imaging into augmented reality technologies (eg, Microsoft HoloLens).

## Conclusions

This study shows that CR imaging speeds up and improves the understanding of complex anatomical situations compared with CT. Surgeons perceive CR imaging as a helpful tool that not only promotes their anatomical understanding but may also enhance preoperative and intraoperative decision making. Additional studies are needed to further explore the benefits of CR imaging in the clinical setting.

## 

## Notes

Supplement. **eFigure.** Likert Plot of the Responses to the General Assessment Questionnaire **eTable 1.** Patient Case Questions **eTable 2.** Case Assessment Questionnaire **eTable 3.** General Assessment Questionnaire **eTable 4.** Results of Objective Assessment According to the Type of Surgeon **eTable 5.** Ratings of the Case Assessment Questionnaire According to Different Categories and Type of Surgeon
