# Supplementary material for: Comparison of Cinematic Rendering and Computed Tomography for Speed and Comprehension of Surgical Anatomy
Source: JAMA Surg. 2019 May 29;154(8):738–44. doi: 10.1001/jamasurg.2019.1168 (PMC6705138; doi:10.1001/jamasurg.2019.1168)
Supplement: Supplement. — eFigure. Likert Plot of the Responses to the General Assessment Questionnaire eTable 1. Patient Case Questions eTable 2. Case Assessment Questionnaire eTable 3. General Assessment Questionnaire eTable 4. Results of Objective Assessment According to the Type of Surgeon eTable 5. Ratings of the Case Assessment Questionnaire According to Different Categories and Type of Surgeon [file jamasurg-154-738-s001.pdf]

Supplementary Online Content

Elshafei M, Binder J, Baecker J, et al. Comparison of cinematic rendering and computed tomography for speed and comprehension of surgical anatomy. *JAMA Surg*. Published online May 29, 2019. doi:10.1001/jamasurg.2019.1168

eFigure. Likert Plot of the Responses to the General Assessment Questionnaire.

eTable 1. Patient Case Questions.

eTable 2. Case Assessment Questionnaire.

eTable 3. General Assessment Questionnaire.

eTable 4. Results of Objective Assessment According to the Type of Surgeon.

eTable 5. Ratings of the Case Assessment Questionnaire According to Different Categories and Type of Surgeon.

This supplementary material has been provided by the authors to give readers additional information about their work.

eFigure. Likert Plot of the Responses to the General Assessment Questionnaire.

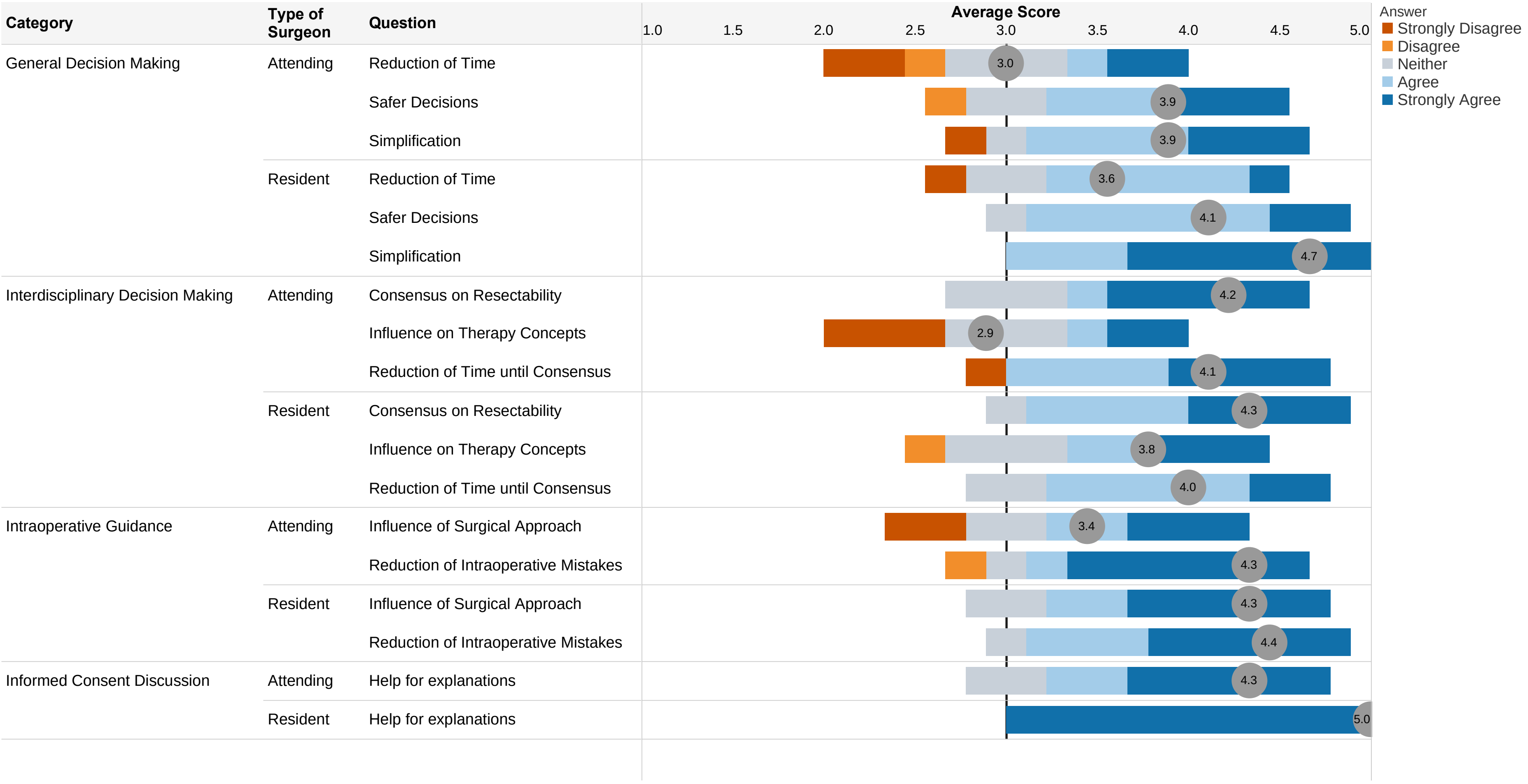

©Elshafei M et al. *JAMA Surgery*.

eTable 1. Patient Case Questions	
**Case No.**	**Question**
Test 1	*Does the tumor in the pancreas head have contact to the superior mesenteric artery?*
Test 2	*Does the ileocolic artery cross the ileocolic vein above or below the origin of the ileocolic vein?*
1	Does the lesion in the pancreas head contact the superior mesenteric vein?
2	Show the first jejunal branch of the superior mesenteric vein.
3	Show the right hepatic artery.
4	Is the right hepatic artery dorsal of the portal vein?
5	Show the bifurcation the right hepatic artery.
6	Does the azygos vein connect at the height of the aortic arch?
7	Does the tumor have contact to the vena cava?
8	What unusual anatomy of the kidney is present in this patient?
9	Does the azygos vein connect to the superior vena cave above or below the tracheal bifurcation?
10	Show the origin of the left hepatic artery.
11	What abnormality is present in the liver contour? Which rib lies next to it?
12	Show the arterio-biliary fistula.
13	Show the bifurcation of the right hepatic artery in the anterior and posterior pedicle.
14	Is the tumor supplied by vessels from the left hepatic artery?
15	Is the superior mesenteric vein compressed by the tumor?
16	Which vessels have contact with the lesion in the pancreas head?
17	Which special liver vein anatomy is present in this patient?
18	Show the two liver lesions at the anterior and posterior pedicle of the portal vein.
19	Which lies closer to the esophageal hiatus, the inferior vena cava or the abdominal aorta?
20	Show the arterial supply of the left liver lobe?
21	Show the confluence of the left gastric vein.
22	Demonstrate the portal vein from its confluence to its bifurcation into anterior and posterior pedicle.
23	Show the arteries in segments IV a and b.
24	Is the umbilical vein recanalized?
25	Does the right portal vein have an anterior and posterior pedicle?
26	Which artery supplies the hepatocellular carcinoma?
27	Does the tumor in the right liver have contact to the right liver vein?
28	Which portal vein pedicle is connected to the hepatocellular carcinoma?
29	Show the lesion in the mesentery of the small intestine.
30	How is the right liver supplied with blood?
31	Show the middle colic artery.
32	How is the liver supplied with blood?
33	Show the common femoral artery. Where does the profunda femoris artery arise?
34	Which artery supplies the left hepatic lobe?
35	Which special vascular anatomy is present in this patient's upper abdomen?
36	Which hepatic segment was resected?
37	Show the closest approach of the left ureter and inferior mesenteric vein.
38	Show the right pulmonary artery and vein.
39	Show the termination of the middle hepatic vein.
40	Where does the superior gluteal artery arise from the internal iliac artery?

©Elshafei M et al. *JAMA Surgery*.

eTable 2. Case Assessment Questionnaire.

The use of Cinematic Rendering is beneficial for the:

SAQ 1 – General comprehension of patient anatomy?				
Strongly disagree	Disagree	Neither	Agree	Strongly agree

SAQ 2 – Comprehension of vascular anatomy?				
Strongly disagree	Disagree	Neither	Agree	Strongly agree

SAQ 3 – Comprehension of parenchymal organ anatomy?				
Strongly disagree	Disagree	Neither	Agree	Strongly agree

SAQ 4 - Comprehension of positional relationship of organs/tumor/vessels?				
Strongly disagree	Disagree	Neither	Agree	Strongly agree

**eTable 3. General Assessment Questionnaire.**					
**General Decision Making**					
CR could help reduce the time needed for therapeutic decisions.					
	Strongly Disagree	Disagree	Neither	Agree	Strongly Disagree
CR could improve the subjective sense of security of therapeutic decisions.					
	Strongly Disagree	Disagree	Neither	Agree	Strongly Disagree
CR could help improve the decision making process in a routine clinical setting.					
	Strongly Disagree	Disagree	Neither	Agree	Strongly Disagree
**Interdisciplinary Decision Making**					
CR could influence decisions regarding resectability of tumors.					
	Strongly Disagree	Disagree	Neither	Agree	Strongly Disagree
CR could influence which therapy concepts patients receive (e.g. adjuvant or neoadjuvant chemotherapy).					
	Strongly Disagree	Disagree	Neither	Agree	Strongly Disagree
CR could reduce the time needed to find a consensus for interdisciplinary cases.					
	Strongly Disagree	Disagree	Neither	Agree	Strongly Disagree
**Intraoperative Guidance**					
CR could influence the potential surgical approach or the type of surgery performed.					
	Strongly Disagree	Disagree	Neither	Agree	Strongly Disagree
CR could reduce intraoperative complications by improving anatomical comprehension.					

©Elshafei M et al. *JAMA Surgery*.

	Strongly Disagree	Disagree	Neither	Agree	Strongly Disagree
**Patient Informed Consent**					
CR can help with explanations during informed consent discussion.					
	Strongly Disagree	Disagree	Neither	Agree	Strongly Disagree

©Elshafei M et al. *JAMA Surgery*.

eTable 4. Objective Assessment According to Type of Surgeon.						
	**Attendings (n=9)**			**Residents (n=9)**		
	**CR-CT Sequence**	**CT-CR Sequence**	*P-Value*	**CR-CT Sequence**	**CT-CR Sequence**	*P-Value*
Correctness in %						
Assessment 1	98.5 (2.3)	88.2 (6.5)		99.0 (2.1)	85.0 (6.8)	
Assessment 2	91.6 (7.8)	100		88.8 (6.2)	99.3 (2.0)	
Interperiod Difference	6.9 (8.4)	-11.8 (6.5)	<0.001	10.1 (5.2)	-14.3 (6.1)	<0.001
Time in s						
Assessment 1	47.1 (47.5)	72.1 (61.8)		66.1 (59.5)	118.6 (95.3)	
Assessment 2	61.9 (59.3)	42.6 (50.7)		88.0 (75.6)	45.3 (46.3)	
Interperiod Difference	-14.8 (68.6)	29.5 (74.1)	<0.001	-21.9 (84.5)	73.3 (94.4)	<0.001

Data are presented as mean (standard deviation).

©Elshafei M et al. *JAMA Surgery*.

eTable 5. Ratings of the Case Assessment Questionnaire According to Different Categories and Type of Surgeon.							
**Category**	**Type of Surgeon**	Strongly disagree n (%)	Disagree n (%)	Neither n (%)	Agree n (%)	Strongly agree n (%)	p-value*
The use of Cinematic Rendering is beneficial for the comprehension of:							
General Anatomy	Resident	0 (0)	1 (0.3)	11 (3.1)	86 (23.9)	262 (72.8)	<0.001
	Attending	3 (0.8)	8 (2.2)	39 (10.8)	122 (33.9)	188 (52.2)	
Vascular Anatomy	Resident	0 (0)	0 (0)	12 (3.3)	48 (13.3)	300 (83.3)	<0.001
	Attending	3 (0.8)	7 (1.9)	32 (8.9)	97 (26.9)	221 (61.4)	
Parenchymal Anatomy	Resident	0 (0)	2 (0.6)	28 (7.8)	78 (21.7)	252 (70.0)	<0.001
	Attending	4 (1.1)	19 (5.3)	53 (14.7)	117 (32.5)	167 (46.4)	
Spatial Relationship	Resident	0 (0)	0 (0)	13 (3.6)	63 (17.5)	284 (78.9)	<0.001
	Attending	6 (1.7)	9 (2.5)	27 (7.5)	109 (30.3)	209 (58.1)	
Data are presented as number (percentage). *Fisher exact test.							

©Elshafei M et al. *JAMA Surgery*.
